# Study protocol for a randomized, controlled trial comparing the efficacy of two educational interventions to improve inhalation techniques in patients with chronic obstructive pulmonary disease (COPD): TIEPOC Study

**DOI:** 10.7573/dic.212261

**Published:** 2014-04-24

**Authors:** José Leiva-Fernández, Francisca Leiva-Fernández, Rubén L Vázquez-Alarcón, Antonio García-Ruiz, Daniel Prados-Torres, Pilar Barnestein-Fonseca

**Affiliations:** 1 Primary Health Care Centre of Vélez-Sur, Health Area Málaga Este-Axarquía, Vélez Málaga (Málaga), Spain;; 2 Multiprofessional Family and Community Medicine Teaching Unit of Primary Care Trust Málaga, Málaga, Spain;; 3 Chair of Health Economics & Rational Use of Drugs, School of Medicine, University of Málaga, Spain

**Keywords:** COPD, inhalation techniques, educational intervention, treatment adherence, self-management, patient empowerment

## Abstract

**Background:**

An appropriate inhalation technique and adherence to treatment are both critical determinants of the success of chronic obstructive pulmonary disease (COPD) management. We have observed that up to 75% of patients do not use a successful inhalation technique. Knowledge evaluation and frequent reassessment of inhaler use, together with education of patients and healthcare professionals, can significantly improve the benefits that patients with COPD will derive from inhaler therapy. The objective of this study is to test the efficacy of two educational interventions to improve inhalation techniques in patients with COPD.

**Methods:**

Multicenter randomized controlled trial with 296 patients diagnosed with COPD selected by a non-probabilistic method of sampling from seven Spanish Primary Care Centers. The patients will be divided into three groups by block randomization. The three groups are: 1) control; 2) Intervention A; and 3) Intervention B. The control group will comprise patients with no explanations or written information; the Intervention A group will comprise patients to whom we give written information only; and the Intervention B group will comprise patients to whom we give written information plus instructor training. Every patient in each group will be visited four times during the year of the study at the health centers.

**Discussion:**

Our hypothesis is that the application of educational interventions (A or B) in patients with COPD who use inhaler therapy will increase the number of patients who perform a correct inhalation technique by at least 25%. We will evaluate the effectiveness of these interventions on patient inhalation technique improvement, where feasible within the context of clinical practice.

## Background

Chronic obstructive pulmonary disease (COPD) is a substantial disease burden worldwide [1]. COPD is the fourth most common global cause of death. Its prevalence is expected to increase and is primarily related to tobacco smoking, affecting 5–10% of the adult population [2–4]. It is characterized by airflow limitation or airway obstruction that is only partially reversible, and is usually relentlessly progressive in nature. For patients with COPD, inhalation therapy is the main treatment. Inhalers are the vehicles for the effective administration of medication. They allow high lung deposition of the drug and minimize systemic adverse drug reactions [5]. Patients often do not get the full value of their inhaled medications because they use their inhaler incorrectly. When technique is markedly flawed, suboptimal outcomes typically result [6]. Some of the consequences of poor inhaler technique include reduced therapeutic dosing and disease stability, which can lead to increased morbidity, decreased quality of life, and a high burden on the healthcare system [7].

The fact that patients with COPD are usually older persons means that they generally have more difficulties effecting successful use of inhalers, and this can be related to tremors, coordination, and visual acuity. Increasing age is significantly associated with an increased risk of inhaler misuse, and cognitive deficits may account for some of the increase seen with disease progression [7].

Although safety and efficacy are the primary factors determining the choice of an inhaler device, there is a need to increase knowledge and optimize clinical use of therapeutic aerosols. Since the inhaler technique required for different types of device varies greatly, it seems sensible to prescribe a single type of device for the different drugs required for the patient. However, this is not always possible, and so different drug and delivery device combinations are prescribed; therefore, it is important to ensure the patient can use them [8–10]. In previous studies we demonstrated that over 80% of patients do not perform a correct inhalation technique [11]. A study of 120 patients (60 COPD; 60 asthma) showed that while 98% of the COPD patients said that they knew how to use inhaler devices, only 5 of the 60 COPD patients (8.3%) performed every step correctly [12]. Other studies show a similar percentage of error [13–15]. Further, many inhalers are complicated to use and some require up to eight steps [16].

There is a wide variety of different inhalers; they can be broadly classified into pressurized metered-dose (pMDI), dry powder (DPI), breath-actuated metered-dose (BA-MDI) and soft mist inhalers. Table 1 describes the percentages of mistakes, step by step, seen by Melani A et al. [17,18] and from our own unpublished data. pMDIs are widely prescribed as they are cheap and can deliver a wide variety of medications for asthma or COPD [19]. A previous study demonstrated that only 79% of patients could use a pMDI correctly after expert training [20]. Efficient use of a pMDI requires coordination between simultaneous inhalation and device actuation, a slow and continuous inspiratory flow rate during inhalation, followed by a breath hold of at least 10 seconds. Patients frequently fail to exhale fully before inhalation. Other common errors are high inspiratory flows, not shaking the device before use, and stopping inspiration when the spray reaches the throat [4,9,16]. Sometimes patients put the mouthpiece into their mouths, which prevents them from getting a good inhalation. It has been demonstrated that if patients purse their lips closely around the mouthpiece and inhale, this allows air from around the mouthpiece to enter, increases the airflow, and moves the medication further into the lungs [21]. Other drawbacks of pMDI are that some of them contain environmentally unfriendly propellants and most of them provide no dose counter, so patients do not know the number of doses remaining and may continue to use the device when it is empty [15,22].

There are a number of DPIs currently available. Some of these devices are single dose, and require the loading of a capsule containing the drug in powder form. The main advantage is that there is no need for an aerosol propellant or coordination between actuation of the device and inhalation. In these devices the most common errors are: not placing the device correctly, exhaling into the mouthpiece, low inspiratory flows, and incorrect breath-holding. The most critical mistakes may be exhalation into the mouthpiece and low inspiratory flow, because the humidity and inadequate flow reduce the amount of drug released and its ability to reach the lungs [22,23].

The National Institute for Health and Care Excellence (NICE) and the Global Initiative for Chronic Obstructive Lung Disease (GOLD) guidelines recommend that prior to prescription of a new inhaler for a patient with COPD, the patient should receive training and education in the use of the device. Both guidelines also advise that inhaler technique should be regularly assessed at each clinic visit [24,25]. The quality of the initial instruction is of utmost importance for the outcome of inhalation therapy. Written instructions alone are insufficient in teaching correct inhalation techniques. Verbal instruction and technique assessment and reassessment are essential for patients to achieve a correct technique [26]. The assumption that healthcare professionals can be relied on to provide patient instruction is called into question by several studies, suggesting that the knowledge and skills of those providing instruction are less than optimal. Most studies indicate that only approximately half of healthcare professionals know how to use an inhaler or demonstrate correct technique [7]. To acquire the skills for using the inhaler devices, healthcare professionals and patients must be adequately educated and trained. Bosnic-Anticevich et al. reported that practical demonstration, in addition to verbal and written instruction, is much more effective in improving technique compared with verbal and written instruction alone [27], but this is a small study in patients with asthma and COPD. Further studies must be conducted to reinforce these conclusions.

The aim of the present study is to test the efficacy of two educational interventions (written only and written and verbal instruction) to improve inhalation techniques in patients with COPD.

## Methods

This study has been approved by the Ethical Committees of Primary Care Trust Málaga (December 21, 2010), the Health Area Málaga Este-Axarquía (February 16, 2010) and by the Regional Clinical Assays Committee (January 25, 2011). The protocol follows the guidelines of the Declaration of Helsinki and Good Clinical Practice.

### Participants

A total of 296 patients with COPD, selected by a non-probabilistic consecutive sampling method from seven Spanish Primary Care Centers, will participate in the study. The inclusion criteria will be: confirmed COPD diagnosis in the clinical record (clinical and/or spirometry criteria following SEPAR or GOLD guidelines), belong to the selected Primary Care Centers in the Málaga area, to have been prescribed inhalation therapy (main devices: Handihaler, Turbuhaler, Accuhaler and pMDI), agreement to participate in the study with written informed consent. Exclusion criteria will be: diagnosis of other respiratory conditions which are not included in the COPD definition (bronchiectasis, asthma or cystic fibrosis) and cognitive impairment problems registered in the clinical record (dementia, Alzheimer’s disease, Parkinson’s disease, cognitive decline). All these criteria will be reviewed in the patients’ clinical records.

### Sample size

This has been calculated to detect a difference of 25% in correct inhalation technique between groups (Intervention A or B vs control), with a statistical power of 80% and a confidence level of 95%, assuming a percentage of expected losses of 40%. The final sample size is 296 patients with COPD who meet the inclusion criteria mentioned above.

### Design

**Figure 1.**
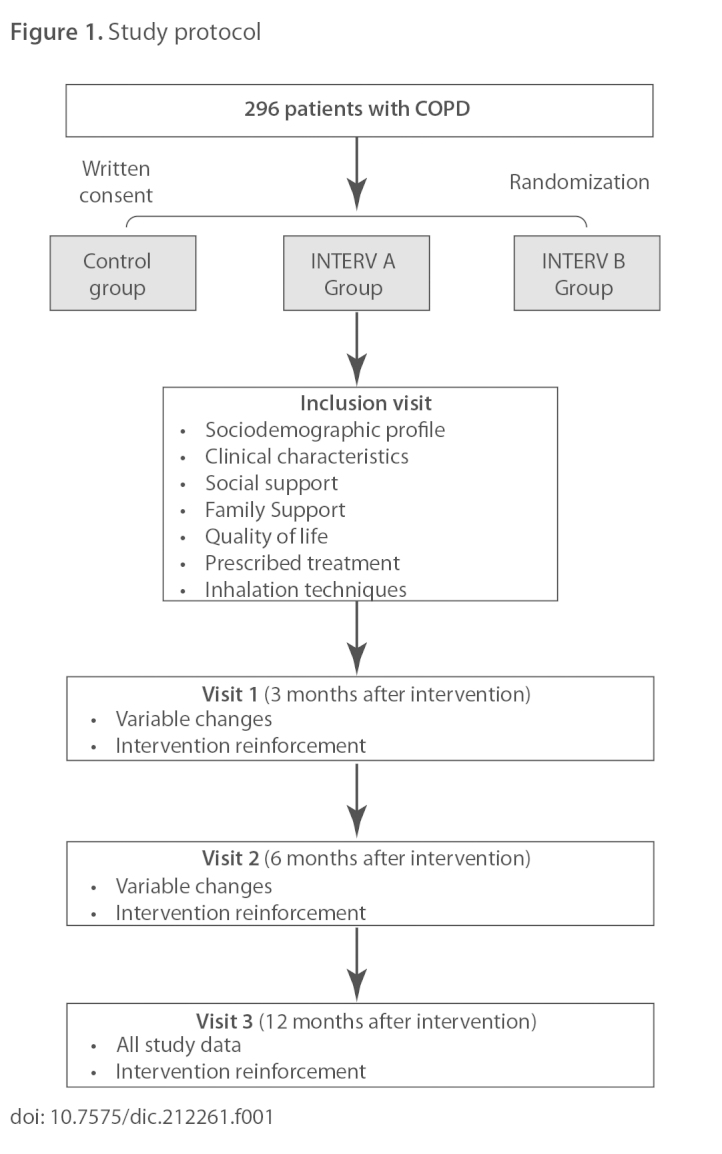
Study protocol doi: 10.7575/dic.212261.f001

Multicenter randomized controlled trial with 1 year of follow-up and four visits (Figure 1). The patients will be divided into three groups by block randomization: control, Intervention A (written information only) and Intervention B (written information and verbal instruction).

### Setting

The study will take place in Primary Care Centers in Malaga, Spain.

### Outcomes

*Primary outcome*: Performance of correct inhalation technique. This will be measured using a template designed following SEPAR (Sociedad Española de Neumología y CirugíaTorácica) guidelines [16] where the interviewer will write down any mistakes. It will be considered a good technique when there are no mistakes registered in the template.

*Secondary outcome:* Participants will be evaluated using inhaler peak flow, functional status (spirometry), mental-cognitive status (mini-mental state examination), dyspnea with the Baseline Dyspnea Index (BDI) [28] and Modified Medical Research Council (MMRC) scale [29]; questionnaires on quality of life (St George’s Respiratory Questionnaire [30], and EuroQoL-5D [31]) and the clinical progress of illness (SeguiEPOC questionnaire) will be recorded.

*Independent* v*ariables:* Independent variables will be: sex, age, educational level, smoking history, comorbidity, COPD severity grade (according to GOLD guidelines [24]), prescribed medication, type and number of devices, family support (Family Apgar Test [32]), and social support (DUKE-UNC Test [33]).

## Intervention

We have designed two educational interventions:

*Intervention A: Written information about inhalation techniques* We will give a leaflet of instructions containing pictures and describing the correct inhalation techniques for the main inhaler devices that can be found in our area: Handihaler, Turbuhaler, Accuhaler and pMDI. The patients included in this group will be asked to show how they use their inhalers and the interviewer will write down the mistakes in a template. When the inhalation technique has been performed, the interviewer will give the leaflet to patients and will invite them to read it and to identify differences between the steps of the correct inhalation technique (leaflet) and the one they have performed.

In the follow-up visits we will ask the patients about the difference between the correct way of performing the inhalation technique shown on the leaflet and how they have been using their inhaler.

*Intervention B: Written information about inhalation techniques and instructor training*. We will give written information about the inhalation technique to the patient (leaflet described above) and we will train the patient in the correct inhalation techniques. The training of instructors in the use of inhaler devices has been carried out at the Pediatrics Pneumology Service of Hospital MaternoInfantil (Málaga). We will perform training in three steps:

– Patients will be asked to show how they use their inhalers, using a variety of placebo inhalers.
– When the patient has given the demonstration, the trainer will ask about the problems and self-perceived mistakes with the technique.
– The trainer will demonstrate the correct technique. Each device will be used and its technique will be explained step by step. The importance of following the correct technique every time the patient uses the inhaler device will be emphasized.

Finally the patients can ask questions and they will practice the techniques until they do it correctly or until the patient becomes tired.

In the follow-up visits, we will review the inhalation technique and we will correct any mistakes or clear up any doubt as explained previously. The objective here is that the patients identify their own mistakes, and if they cannot, to remind them of the proper technique by giving as many demonstrations as necessary.

### Recruitment

Patients who meet the requirements described above will be contacted using their health center records. They will be invited to participate in the study after a brief explanation by telephone, and they will receive an appointment in the health center. At this first appointment (inclusion visit) patients will receive more detailed information about the study and if they agree to participate, they will sign the written consent.

At this point, they will be divided into three study groups: Intervention A, Intervention B and a control (Figure 1). The randomization will be made using the block randomization technique and applied separately at each study center. The blocks consist of six patients, two subjects per arm; or three patients, one subject per arm (Intervention A, Intervention B and control). The blocks will be marked with a number and they will be chosen at random to create the allocation sequence using a series of random numbers generated by a Microsoft Excel 2003 program. The person responsible for assigning patients to this group will make contact by phone. Because the patients will be from various health centers, the presence of the two interventions and control subjects in all the health centers will be guaranteed.

After randomization, all the study data will be recorded and the performance of correct inhalation techniques will be measured in all groups (inclusion visit). In the case of the control group, the inhalation techniques will be tested by asking patients about how they used their inhalers and the interviewer will write down the mistakes in the template. The interviewer will only correct the critical mistakes previously agreed by the research team and all interviewers who participate in the study. In the follow-up visits the subjects will be invited to perform the inhalation technique and the mistakes will be written down. The appointments last approximately 20–30 minutes, depending on the study arm.

### Follow-up

All the patients will undergo the same follow up: four visits within 1 year.

#### Control group:

Inclusion Visit: all the study data will be recorded and the inhalation technique will be tested.

Visit 1 will take place 3 months after the inclusion visit. Primary and secondary outcomes will be measured (excluding spirometry and quality of life).
Visit 2 will take place 6 months after the inclusion visit. Primary and secondary outcomes will be measured (excluding spirometry and quality of life).
Visit 3 will take place 12 months after the inclusion visit. All the study data will be recorded.

#### Intervention A group:

Inclusion Visit: all the study data will be recorded and the inhalation technique will be tested.

Visit 1 will take place 3 months after the inclusion visit. Primary and secondary outcomes will be measured (excluding spirometry and quality of life) and correct inhalation techniques using the designed leaflet will be encouraged.
Visit 2 will take place 6 months after the inclusion visit. Primary and secondary outcomes (excluding spirometry and quality of life) and correct inhalation techniques using the designed leaflet will be encouraged.
Visit 3 will take place 12 months after the inclusion visit. All the study data will be recorded and correct inhalation techniques using the designed leaflet will be encouraged.

#### Intervention B group:

Inclusion Visit: all the study data will be recorded and the inhalation technique will be tested.

Visit 1 will take place 3 months after the inclusion visit. Primary and secondary outcomes will be measured (excluding spirometry and quality of life) and encouraging correct inhalation techniques using the leaflet and the training with the monitor focusing on motivational aspects to enhance the individual skills of the patients will be individually applied.
Visit 2 will take place 6 months after the inclusion visit. Primary and secondary outcomes will be measured (excluding spirometry and quality of life) and encouraging correct inhalation techniques using the leaflet and the training with the monitor focusing on motivational aspects to enhance the individual skills of the patients will be individually applied.
Visit 3 will take place 12 months after the inclusion visit. All the study data will be recorded and encouraging correct inhalation techniques using the leaflet and the training with the monitor focusing on motivational aspects to enhance the individual skills of the patients will be individually applied.

A patient will be considered as being lost to follow-up if:

– The patient has not attended two follow-up visits
– The patient has declined to continue in the study
– The devices considered in the study have been withdrawn

### Statistical Analysis

A descriptive statistical analysis will be performed for all the study variables. We will calculate the mean, median and standard deviations for quantitative variables, and the absolute and relative frequency for qualitative variables. The 95% confidence interval will be applied. A baseline comparison will be made between all groups to analyze the comparability using the chi-square test or ANOVA.

The analysis will be made following an intention-to-treat procedure [34].

The between-group comparison for the primary outcome will be explored using the chi-square test (Intervention A vs control; Intervention B vs control; Intervention A vs Intervention B).The Relative Risk Reduction (RRR), the Absolute Risk Reduction (ARR) and the Number Needed to Treat (NNT) will be calculated. Inferences for the secondary outcomes will be made using an analysis of variance (ANOVA) or chi-square test. Each group will be analyzed separately.

Finally, a logistic multivariate regression model will be performed for the primary outcome (performance of correct inhalation technique [yes/no]), considering the intervention as the predictive variable and the rest of the independent measures as the possible modifying factors.

We will use a 5% significance level (*p*=0.05). The SPSS 15.0 and Stata 11.1 statistical packages will be used to run the analysis.

## Study limitations

One limitation of this study is the selection bias due to the missing data. In order to decrease this bias, we will apply several strategies:

- An increase of 40% in the sample size (expected losses)
- Three phone calls on different days and times for unreachable patients
- Rescue appointments for the non-attendees to visits (three different appointment dates)

In addition we take into account the possible contamination between the control and the intervention groups because of the relationship between subjects in their daily life (neighborhoods, relatives, social networks or associations). However, in another educational intervention study performed with obese patients in our area we did not find a significant level of this effect [35]. We also believe that the intervention characteristics (several steps and individual visits) that we have described will have little influence on contamination between groups.

In order to standardize interventions they will be performed by two professionals trained in communication, the knowledge of disease, and inhalation techniques of the different inhaler devices. Furthermore, we have designed a manual for the researchers where we explain the working plan, the different parts of the intervention, and the protocol in order that they know what they have to measure each time and the details to assess each variable included in the study. In this way, the procedure can be replicated elsewhere.

## Discussion

Previous to this study, we designed and developed a multifactorial intervention to improve adherence in COPD, the ICEPOC study [36]. This study allowed us to analyze the motives and barriers that these patients experience in complying with the recommended medication regimens. The multifactorial intervention included training about inhalation techniques and we could see that up to 75% of patients did not perform them correctly. After the intervention we found that 17% still did not perform a correct inhalation technique [11] (52% of the patients in the intervention group enhanced their inhalation technique). But this study did not have a control group. So we designed a randomized controlled trial to define the role of the educational interventions over inhalation techniques. A randomized controlled trial (RCT) is regarded as the gold standard for assessing the effectiveness of treatment. It has the power to eliminate a variety of alternative explanations for changes in health-related measures over time, permitting the researcher to reasonably conclude that the intervention itself caused the changes.

The NICE and the GOLD guidelines recommend that prior to prescription of a new inhaler for a patient with COPD, the patient should receive training and education in the use of the device. Both guidelines also advise that inhaler technique should be regularly assessed at each clinic visit [24,25]. Written instructions alone are insufficient in teaching correct inhalation techniques. Verbal instruction and technique assessment and reassessment are essential for patients to achieve a correct technique [26].

Our hypothesis is that the application of educational interventions (Intervention A or Intervention B) in patients with COPD with inhaler therapy is going to help up to 25% of patients, who have been misusing their inhalers, improve their inhalation technique. These interventions are feasible to implement in the clinical practice context. With this strategy we are trying to promote patients’ autonomy and responsibility about their disease, achieving in this way a greater improvement on their health outcomes and increasing clinical effectiveness in our daily practice.

## Figures and Tables

**Table 1.** Inhalation technique percentage of mistakes.

	**Single-DPI**	**Multiple-DPI**	**pMDI**
Removing the cap	x	x	x
Correctly inserting capsule	x	NA	NA
Shaking inhaler	NA	NA	xx
Loading the device	x	x	NA
Emptying the lungs (avoiding exhaling into the inhaler for DPI)	xxx	xxx	xxx
Placing the mouthpiece in the mouth, closing lips around it, and avoiding any obstruction with the tongue	x	x	x
Holding inhaler upright during actuation	NA	NA	x
One inhalation for actuation	NA	NA	x
Inhaling with the maximum inhalatory force from the start	xx	xx	NA
Activating the inhaler during the first half of inhalation	NA	NA	xxx
Slowly inhaling while activating the inhaler	NA	NA	xxx
Continuing to fill the lungs completely	NA	NA	xx
Holding breath for 10 seconds	xxx	xxx	xxx
Repeating the inhalation	x	NA	NA
Checking whether the capsule is empty	x	NA	NA
Closing the inhaler	x	x	x

**Abbreviations**

Single-DPI: single-dose dry powder inhaler; Multiple-DPI: multiple-dose powder inhaler; pMDI: pressurized metered-dose inhaler; x: occasional mistake; xx: not-infrequent mistake (up to 20%); xxx: common mistake (up to 50%); NA: not applicable.

Modified from Melani A et al. [17,18] and from our own unpublished data.

doi: 10.7575/dic.212261.t001

## Abbreviations

ANOVA: analysis of variance;
ARR: absolute risk reduction;
BA-MDI: breath-actuated metered-dose inhaler;
BDI: Baseline Dyspnea Index;
COPD: chronic obstructive pulmonary disease;
DPI: dry powder inhaler;
GOLD: Global Initiative for Chronic Obstructive Lung Disease;
MMRC: Modified Medical Research Council scale;
NICE: National Institute for Health and Care Excellence;
NNT: number needed to treat;
pMDI: pressurized metered-dose inhaler;
RCT: randomized controlled trial;
RRR: relative risk reduction;
SEPAR: Sociedad Española de Neumología y CirugíaTorácica;
SPSS: an IBM statistics software package.
